# Systematic Literature Review on Donkeys (*Equus asinus*): Husbandry and Welfare in Europe

**DOI:** 10.3390/ani15192768

**Published:** 2025-09-23

**Authors:** Naod Thomas Masebo, Beatrice Benedetti, Maria Gaia Angeloni, Leonie Lee, Daniele Bigi, Barbara Padalino

**Affiliations:** 1 Department of Agricultural and Food Sciences, University of Bologna, Viale Fanin 46, 40127 Bologna, Italy; naodthomas.masebo2@unibo.it (N.T.M.); beatrice.benedetti7@unibo.it (B.B.); mariagaia.angeloni@unibo.it (M.G.A.); daniele.bigi@unibo.it (D.B.); 2 Equined, Geelong, VIC 3220, Australia; leonie@equined.com.au; 3 Faculty of Science and Engineering, Southern Cross University, Military Road, Lismore, NSW 2480, Australia

**Keywords:** dairy donkeys, donkey for intervention therapies, donkeys used for meat production, dental disorders, feeding, hoof disorders, housing

## Abstract

The number of donkeys in Europe has significantly declined in recent decades due to mechanization; however, recently, the demand for donkey milk and other purposes has led to a slight increase in their population. However, information on how they are kept and managed, and their welfare is limited. This review aimed to explore the husbandry, management, and welfare of donkeys (*Equus asinus*) across European Union member states, Switzerland, and the United Kingdom. The Preferred Reporting Items for Systematic Reviews and Meta-Analyses (PRISMA) technique was used. The search was conducted using Scopus and Web of Science, identifying 797 records that were screened using titles, keywords, and abstracts, resulting in 78 retained records. An additional 19 records were identified using snowballing and experts’ suggestions, bringing the total to 97. Dairy donkeys have been studied mainly in Italy, and there they are usually managed under extensive to semi-intensive husbandry systems. Donkeys involved in human intervention therapies are generally managed semi-intensively. Based on the literature, most donkeys are provided with shelter and outdoor access, and this can be with or without pasture, except the free-range donkeys that graze year-round. Health and management-related issues (e.g., obesity, dental disorders, and hoof disorders) could be overlooked, potentially compromising their welfare. The feeding management of donkeys is generally traditional and poorly studied, relying mainly on forages supplemented with concentrates. Most donkeys suffer from overweight/obesity except for lactating donkeys, which are often underweight. This may indicate unbalanced feeding practices. Improved understanding of housing and feeding management is essential for establishing evidence-based welfare guidelines tailored to the donkeys’ species-specific needs.

## Simple Summary

In the past, donkeys were commonly used in Europe for farming and transportation. However, with the rise of machines, their numbers declined. Recently, interest in donkeys has grown again, especially for their milk and other uses, leading to a small increase in their population, but information on their quality of life is limited. This review looked at how donkeys are managed across the European Union member states, plus Switzerland and the United Kingdom, focusing on their housing, feeding, and overall welfare. The Preferred Reporting Items for Systematic Reviews and Meta-Analyses (PRISMA) technique was used as the methodology for this review. The search was performed in the Scopus and Web of Science bibliometric databases. In the investigated countries, dairy donkeys are kept under various management systems ranging from free-range to more controlled (semi-intensive) environments. Those used for other purposes, such as meat production, are provided with various forms of shelter and outdoor access, with varying pasture availability. Feeding practices for donkeys are not well researched. Most donkey owners rely on traditional feeding methods, generally providing hay and some concentrated feed. Common issues, like poor dental or hoof care, can go unnoticed, affecting the donkeys’ health and well-being. The review highlights the need for more research on minimal welfare standards for donkeys, based on their species-specific needs and the purpose for which they are kept.

## 1. Introduction

Donkeys (*Equus asinus*) were one of the earliest domestic animals and were crucial to the growth of the human population. Donkeys continue to be considered as working animals today, supporting millions of people in many developing countries [1]. Before World War II, donkeys were employed in a variety of tasks in Europe, including human transportation and agricultural tasks. Due to technological development and agricultural mechanization, the use of the donkey has significantly decreased, leading to a decline in their number [1,2]. Indeed, the number of donkeys raised in Europe (i.e., the full continent) has dropped by more than 80% since the middle of the 20th century and by 50% between 1994 (total *n* = 967,057 heads) and 2014 (total *n* = 514,591) [1]. However, this number has somewhat recently increased as a result of the rising demand for donkey milk and other purposes [1,3]. According to FAO estimates, in 2022, there were 100,208 donkeys living on the European continent [4]. Although not all nations have mandatory donkey registration, and it is likely that this number is underestimated [5], making accurate donkey population estimates difficult to obtain [6].

Donkeys are naturally adapted to an extreme environment with low resource inputs. The appropriate species-specific management of donkeys is important to prevent management-related problems, including conditions such as obesity and laminitis [7]. Currently, different donkey breeds are raised for a variety of purposes, primarily for dairy production, companion animals, assisted therapy, tourist trekking, and as livestock guardians. Additionally, draught donkeys are kept on small farms instead of tractors [8] and are used to maintain the soil and landscapes [1]. Certain donkey breeds are raised for their meat, especially in the southwestern region of Europe [1]. According to Kugler et al. [9], there are approximately 60 donkey breeds in Europe, with the greatest presence in France, Italy, and Spain. The increase in the donkey population in Europe over the last two decades has consequently led to an increase in the number of native donkey breeds in various countries. For example, in Italy, the number of animals belonging to the native eight breeds (i.e., Amiata, Asinara, Martina Franca, Pantesco, Ragusano, Romagnolo, Sardo, and Viterbese) in the stud book increased fourfold between 2010 and 2020 [10].

However, information on the different types of husbandry systems and management practices is scarce. Similarly, not much is known about the welfare level of these animals. For instance, donkeys, especially those not used as working animals, usually live until old age (≥30 years), and some of the management-related disorders, including dental and hoof issues, could be overlooked, leading to their poor quality of life. Conversely, donkeys used as working animals may experience ongoing exhaustion, and their management could also lead to poor health and welfare [7]. Therefore, using a deep analysis of the literature, the current review aimed to document the most common donkey (*Equus asinus*) husbandry systems and management practices in European Union (EU) member states (MSs), plus Switzerland and the United Kingdom (UK), to discuss their impact on the welfare of donkeys, and to propose best practices for safeguarding them. This review may also provide useful evidence to inform ongoing Equidae (including donkey) welfare, management and, husbandry policy development [3,11,12].

## 2. Materials and Methods

### Literature Search Methods and Selection Strategy

To perform the literature search, the Preferred Reporting Items for Systematic Reviews and Meta-Analyses (PRISMA) method was used [13]. To decide the most appropriate search strings, the authors engaged in discussions and the final strings of keywords were selected after undergoing several trials. This process helped refine the keyword combinations, ultimately leading to the selection of search strings that best aligned with the objectives of the study. To find the relevant records, two databases were used, namely Elsevier©’s bibliometric database, Scopus (https://www.scopus.com/home.uri, accessed 20 June 2025), and Web of Science (https://www.webofscience.com/wos/woscc/advanced-search, accessed 20 June 2025). The final search string keyword combinations are reported in Supplementary Table S1. The pre-filter criteria included publication years from 2005 to 2025, record types (articles, reviews, and book chapters), language (English), scientific fields (veterinary sciences and agriculture), and author affiliations from EU MSs plus Switzerland and the UK (Supplementary Table S2). Those countries were investigated, as the European Commission, Switzerland, and the UK Governments are in the process of issuing new policies for the protection of Equidae, including donkeys. In addition, the European Commission has accepted the government’s role of working horses, donkeys, and mules and acknowledged that more can be done to provide them with better welfare [3,11,12].

The search was conducted based on the title, abstract, and keywords of the documents. These pre-filters were discussed and agreed upon by the authors to capture the most recent evidence published over the past 20 years. This approach is supported by the standard methodology used in several Scientific Opinions of the European Food Safety Authority [14] and in the literature [15,16].

**Figure 1:**
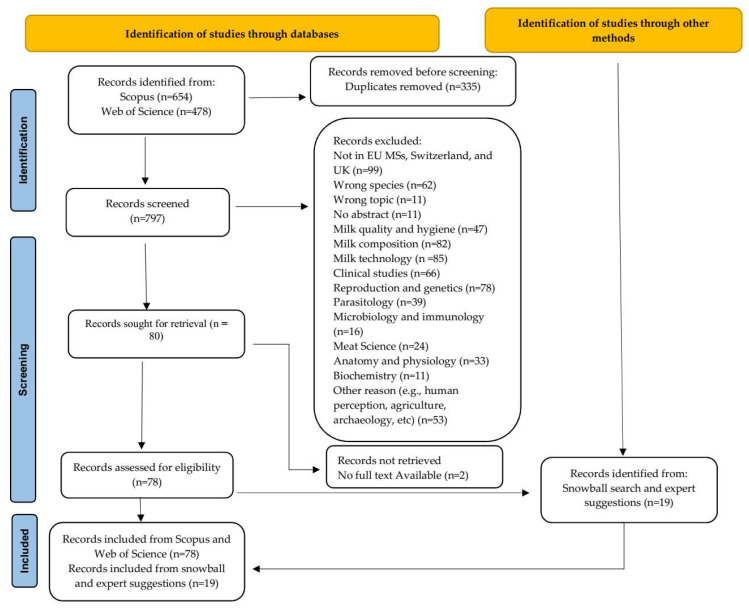
Selection procedure and total number of records included (*n* = 97) in this systematic review, comprising articles identified through Scopus and Web of Science (*n* = 78), and additional records retrieved via snowball search and experts’ suggestion (*n* = 19). The figure also reports the number of excluded records and the exclusion criteria. EU MSs: European Union member states; UK: United Kingdom.

A single spreadsheet (Microsoft Excel^®^, version 16.0, Redmond, WA, USA) was downloaded by each portal and then used to store the generated set of records. In the final dataset, appropriate information was grouped in columns and individual records were arranged in rows. For each entry, specific information was noted, including the title, abstract, year of publication, authors, corresponding author, affiliations, record type, publishing source (e.g., journal title), and keywords. The search revealed a total of 1132 records, and 335 of these were removed because they were duplicates. As a result, 797 records were retained and screened (Figure 1).

After deciding on inclusion and exclusion criteria (Table 1), researchers thoroughly filtered and screened the records by reading the abstracts, keywords, and titles. The records were considered relevant and retained when they focused on husbandry, management, welfare, dental disorder and dental care, and hoof disorder and hoof care of donkeys (*Equus asinus*) in the investigated countries (e.g., some corresponding authors could have been based in an EU MS but the work was performed in another country, such as Ethiopia, leading to exclusion of that record). The senior author (BP) made the final choice in cases where it was not evident whether a document should be included or not.

The majority of the records were excluded because they were not conducted in the countries of interest. The other main causes of exclusion included the studies conducted on donkey milk, focusing on the nutritional quality/benefits, compositions, technological advancement, and microbiological quality, meat and quality of the carcasses without information on husbandry and management. Following the records screening, the full texts of the included articles were downloaded, read, and assessed to extrapolate the necessary information.

After the initial screening, 717 records in total were removed and categorized in line with the exclusion criteria. Only 80 records were kept for full-text reading; however, it was not possible to download two articles, so information was extracted from 78 documents. During this phase, the authors also performed a snowball search by examining the list of references of the retained records. In addition, papers suggested by an expert in the field were also considered. Seventeen records were chosen by snowballing and 2 by expert suggestion, for a total of 19 additional records (Figure 1).

Information regarding husbandry, management, welfare, dental disorders/care, and hoof disorders/care was extracted from the records and summarized. The types of husbandry were defined based on the amount of land (pasture) availability, and the level of human intervention in the management of the donkeys. Hence, they were categorized into ‘intensive’ (10–20 m^2^) with almost no pasture, and animals were entirely housed almost all time, ‘semi-intensive’ (50–250 m^2^), with pasture availability and additional shelter for overnight access, and ‘extensive’ (5000 m^2^ or 2 animals/hectare), with no or very limited human intervention, and entirely pasture-based [17,18,19]. The results are presented based on the use and purpose of the donkey-keeping practices, as housing and feeding arrangements may vary, with dairy donkeys requiring specialized housing and feeding conditions.

## 3. Results

**Figure 2:**
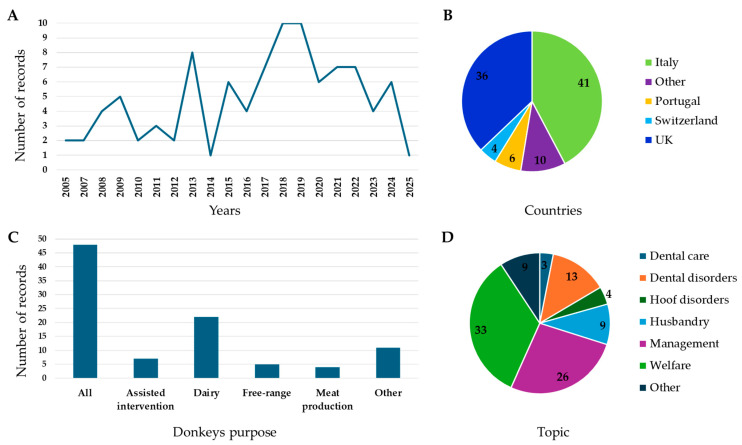
(**A**) Distribution of the number of records selected per publication year (2005–2025); (**B**) distribution of the number of records selected per country of affiliation of the corresponding author. The category “Other” included Belgium, Croatia, Czech Republic, Greece, The Netherland, Ireland, and Slovenia; (**C**) distribution of the number of records selected per donkeys’ purpose. The category “All” included papers talking about donkeys in general while the category “Other” refers to papers talking about working donkeys, companion donkeys, and donkeys in zoos; (**D**) distribution of the number of records selected per topic of the paper. The category “Other” refers to topics such as physiology and health.

Considering the 78 records originally selected and the 19 records selected with snowballing and experts’ suggestion, the number of papers per year has remained more or less stable, with no clear positive or negative trend (Figure 2A). The records were written mainly by authors affiliated with Italian, British, Swiss, and Portuguese Universities (Figure 2B). Most of the records dealt with donkeys in general, followed by papers on dairy donkeys (Figure 2C), while the main topics covered were welfare and management (Figure 2D).

### 3.1. Dairy Donkey

Dairy donkeys are becoming increasingly popular in Europe [20,21,22], particularly in Italy, Spain, Portugal, France, Cyprus, but also in Belgium, the Netherlands, and Romania. The increased popularity of the dairy donkey is due to an increased demand for their milk, valued for its nutritional properties and for having a composition like human milk, making it suitable for children and elderly people who are allergic to other milk types [2,23]. Furthermore, donkey milk is used in cosmetic products [1,2,23,24]. As a result, dairy donkey management is currently shifting from traditional to modern management systems [2]. However, little is known about the impact of the new management systems on donkey welfare and health [25]. Most of the research on milk production and dairy donkey management comes from Italy [21,23,25,26]. This is not surprising as in Italy, 122 donkey farms are officially registered as dairy farms, for a total of around 600 animals [27]. As selection for milk production in donkeys is only just beginning [28], there are no specialized donkey breeds for milk production yet. However, some breeds, namely Ragusano, Martina Franca, and Romagnolo, are more widely used for milk production as these are the larger breeds, and there is a positive correlation between jenny size and milk production [10]. Furthermore, the use of reproductive biotechnologies in donkeys is currently limited. A suitable method for donkey semen cryopreservation would be very valuable for the ex situ management of genetic diversity, but results have not been optimal [29]. Various authors have documented the low fertility obtained with frozen donkey semen using different extenders and amounts of cryoprotectant (CPA) or combinations of CPAs. Studies on embryo transfer (ET) in donkeys have been conducted over the last four decades, but the efficiency of this biotechnology is still very limited. More work still needs to be carried out to develop suitable adaptations of in vivo and in vitro embryo production and transfer protocols to the donkey species [29].

#### 3.1.1. Housing System of Dairy Donkeys

Although limited information is available on dairy donkey housing systems, these animals are generally managed under extensive, semi-extensive and semi-intensive husbandry systems [2,20,21,23,30,31], with housing practices varying widely among farms. Smaller farms tend to keep donkeys in semi-intensive systems while larger dairy farms keep them in intensive systems [22]. In Italy, across 12 farms assessed by Dai et al. [21], all donkeys were managed under a semi-extensive system, characterized by continual access to pasture with constructed shelter. Only two farms had stables to keep the donkeys in overnight. Most of the farms kept donkeys in separate paddocks based on their production characteristics (lactating, dry, stallions). Lactating and dry jennies were kept separately to better manage milk production and feeding. Stallions were housed in groups with females on nearly half of the farms, while they were housed alone on the other half (usually in box stalls) [21]. After weaning, foals are usually taken to a pasture to allow natural grazing behavior, with access to hay mangers, feeders, and a built-in shelter. When pasture quality is poor or insufficient to meet nutritional needs, hay mangers play a crucial role [32].

A modern dairy donkey facility generally contains stables, paddocks, a waiting area (i.e., a holding pen for keeping jennies before they enter the milking parlor), a milking parlor (i.e., a separate place where jennies are brought to be milked), a milk storage room, and a milk collection center. Health check-up boxes are also present for examination or occasional monitoring. Furthermore, a maternity and foaling area is provided to manage complications that may arise before birth. This area is usually 4.0 m in length and 4.2 m in width to allow for adequate room to assist the delivering donkey [20].

**Figure 3:**
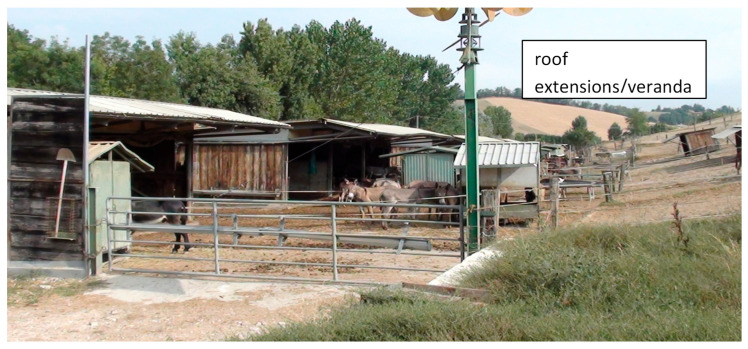
Donkeys kept in groups in paddocks are all provided with a shelter, a feeding point, and a drinking point (Allevamento Montebaducco, Reggio Emilia) (Padalino, picture).

Stables can be enclosed (i.e., fully surrounded by walls and covered with a roof) or open-sided (i.e., having an entire side directly open to the outdoor environment) buildings for housing donkeys indoors [20]. According to Bigi [10], the space allowance should be of at least 4.5 m^2^ per donkey, and the roof should be at least 1 m higher than the tallest animal’s withers. Stables often have an access opening to the paddock (with or without a veranda), allowing donkeys to move freely inside and out. A paddock is a fully fenced outdoor area for donkeys. The fence railing can be constructed of materials, such as steel, timber, or steel mesh. Electric fences are also becoming more common, with tape preferred for visibility [10]. The paddock is usually provided with a constructed or natural shelter, and one or more feeding and watering points [10] (Figure 3). It is also designed to facilitate the milking workflow, with the appropriate placement of materials, fixtures, and fittings (e.g., sheds, troughs, feeders) [20].

**Figure 4:**
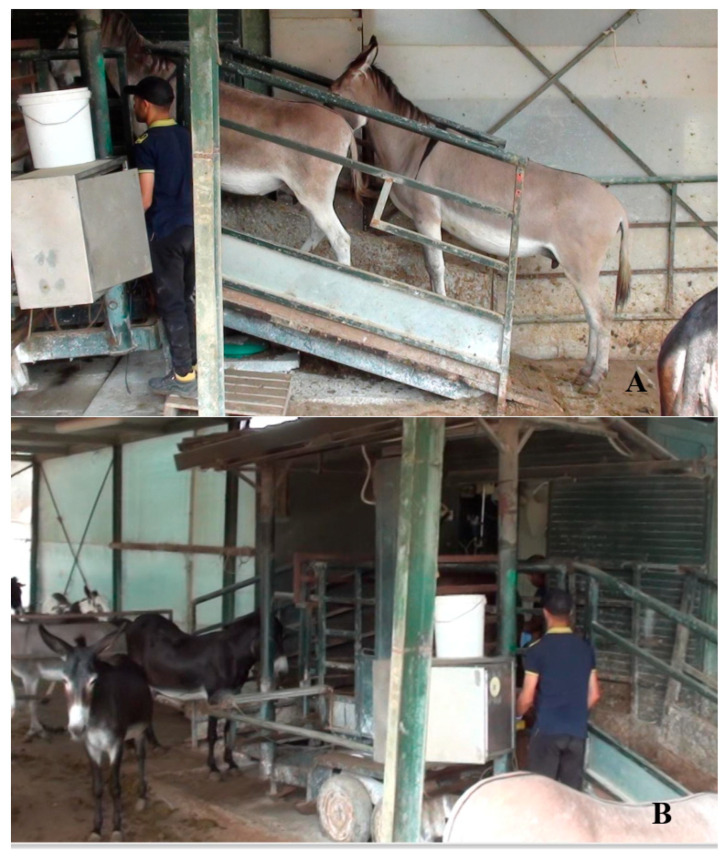
Entrance of a milking parlor on a dairy farm in Reggio Emilia. The donkeys line up single file and go up the ramp to be milked (**A**), then down another ramp to remain in the waiting area (**B**) (Allevamento Montebaducco, Reggio Emilia) (Padalino, pictures).

**Figure 5:**
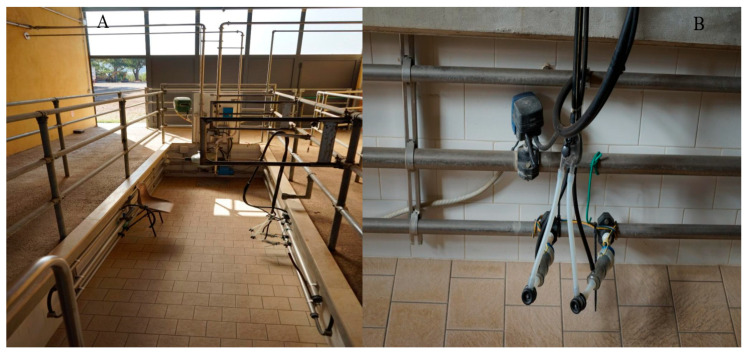
(**A**) Two-station milking parlor and (**B**) automatic milking machine in dairy donkey farm (Bigi, pictures).

Typically, the dairy donkey milking parlor (Figure 4) is a simple modification of a milking setup and arrangement. Each jenny is milked individually, usually using a single milking machine while other donkeys wait their turn. Large dairy donkey farms (i.e., >30 dairy jennies) may have bigger milking parlors with different arrangements (Figure 5). The machine milking method has been utilized in dairy donkeys; however, detailed studies regarding its effects on milk production and udder health are lacking, and further research is needed to better understand the application of the milk machine in the dairy donkey farm and potentially improve conditions [33].

**Figure 6:**
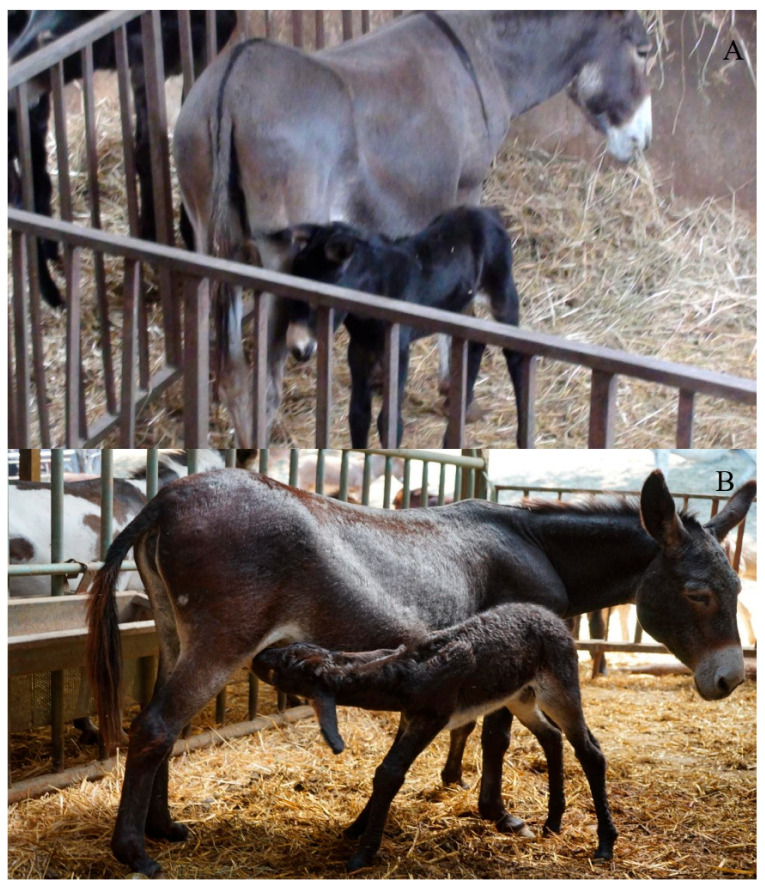
Donkey maternity area usually consists of a fenced indoor area, with a concrete floor and straw bedding. Jennies with newborn foals in the maternity area (**A**); newborn foals suckling the jenny in the maternity area (**B**) (Allevamento Montebaducco, Reggio Emilia) (Padalino and Bigi, pictures).

The maternity area (Figure 6) is typically a pen or a set of box stalls inside the stable. The pen is disinfected before and after each delivery and has enough clean bedding material, usually straw, to minimize the risk of infectious disease [34]. Generally, the depth of the bedding materials should be at least 20–30 cm across the entire floor, and even deeper (up to 40 cm) in cold climates to provide extra insulation [16]. The floor itself is typically fully concrete with rubber mats. The jenny is kept by herself for at least one day to facilitate the bonding with the foal and ensure the foal has colostrum intake [35]. After the first 24 h of life, the jenny and the foal are provided with outside access [36] and are reintroduced into the group. Pregnant jennies are moved to maternity areas/pens a few days before their expected foaling date. This is estimated from the average gestation length typically around 370 days from the last mating and clinical examination of the udder [35]. Ultrasonography may be used alongside clinical signs to refine the prediction. A noticeable enlargement of the udder indicates imminent delivery [36].

During milking, most of the foals are separated from their mother and kept in an adjacent paddock where they can see and/or touch them [21]. Artificial suckling with milk replacers was tested and deemed possible but not regularly provided in the field [37,38]. Foals typically receive their milk straight from the mother [34]. The nutritional requirements of foals have not been fully studied, and only limited information is available about their growth rate. Nursing foals are usually fed easily digestible creep feed, specially formulated for young foals until they reach one month of age, after which they no longer receive the milk from their mother. From this point, complementary feeds are provided until weaning. During this period, constant access to clean water and salt blocks is highly recommended for both foals and dams [39]. The weaning process of foals differs from farm to farm. At some dairy donkey farms, foals remain with their mother, while at others, the weaning process takes place step by step and the oldest foals are weaned first, forming new yearling groups. On certain farms, the weaning process is conducted by removing one jenny at a time, allowing the group to settle down until the next jenny is removed. Typically, foals are weaned when they are between 7 and 12 months old [21].

When bedding is provided for dairy donkeys, it typically consists of straw (wheat, barley, or oat straw) or wood shavings. Wood pellets, rice hulls, paper, sand, and sawdust are less common. The choice of bedding material is relevant for donkeys’ nutritional welfare, because edible bedding materials may represent a risk factor for the development of nutritional disorders. The use of cardboard or paper has been associated with an increased risk of hyperlipemia and colic in donkeys and is, therefore, not recommended [40,41]. Straw bedding is generally recommended as the best option; however, since it can be eaten, straw intake needs to be monitored. Excessive consumption may cause overweight in donkeys and increase the risk of laminitis and impaction colic in donkeys. Appropriate management, including both the type and quantity of bedding, as well as regular cleaning and/or replacement, is crucial, especially in intensive dairy donkeys farming systems where the animals are confined to stalls and/or loose boxes without access to pasture [24].

#### 3.1.2. Feeding Practices of Dairy Donkeys

The welfare of jennies and their foals depends on a precise understanding of the requirements of dairy donkeys during pregnancy and lactation, which is also essential to maintain and improve milk production. For dairy donkeys, lactation is a crucial stage of the breeding cycle during which appropriate care and nutritional requirements must be met. The nutritional requirements of dairy donkeys remain little understood as limited research is available about their energy, protein, vitamin, and nutrient requirements across different stages of production and pregnancy [24]. Salari et al. [42] investigated the daily feed intake of donkeys during maintenance, late pregnancy, and early lactation. Donkeys in the maintenance phase require a slightly higher net energy intake. During late pregnancy, dry matter (DM) intake dropped by 31%, increasing the risk of hyperlipemia and highlighting the need for higher-energy diets during the third trimester. Weight loss in early lactation suggests that the nutritional demands of lactating jennies are not fully met and require further investigation [24]. Most dairy donkey farms do not provide scientifically formulated rations, and feeding practices vary by farm. For example, in Italy, Dai et al. [21] indicated that the feeding of lactating jennies is typically based on pasture and hay (*ad libitum*), plus concentrate (barley, oat, or compound feed), and a mineral supplement. Across different farms, there is generally little variation in the feeding management of lactating versus dry jennies. Cavallarin et al. [23] reported that in Northern Italy, donkey feeding relied on partial grazing combined with *ad libitum* access to farm-produced hay, with animals spending almost 12 h per day on pasture. In Croatia, Ivanković et al. [43] reported that the feeding of lactating jennies consisted of hay and pasture, supplemented with 1.0–1.5 kg/day of concentrates (grains) for Littoral Dinaric jennies, and 1.5–2.0 kg/day for Istrian jennies. Another research by Salari et al. [30] reported that in Italy, jennies were fed mixed hay *ad libitum* and about 2.5 kg/day/head of concentrate.

Therefore, detailed studies are needed to understand the DM requirements of a dairy donkey and their intake. It is suggested that during the first three months of lactation, a donkey should receive approximately 3.3 kg DM/100 kg BW from forage and 1.65 kg from concentrates [24]. There is no information available about donkeys’ protein needs, but it was proposed that the protein needs for a lactating jenny are roughly twice that of the normal maintenance requirement [24]. Similarly, mineral and vitamin requirements are not well established. Although donkeys can survive on a lower level of vitamins and minerals than horses, it has been proposed that the National Research Council’s (NRC) recommendation for the maintenance requirement of vitamins of horses may serve as an appropriate reference for lactating jennies [24,44].

Although donkeys may endure extended periods of water deprivation [45], their water requirements should not be underestimated, as intake depends on workload, type of food (fresh forages versus hay-based diets), working hours, physiological state (i.e., lactation, dry, or pregnant) and environmental conditions [24,45]. Among these factors, lactation is particularly relevant, as it leads to an increase in water intake in all milk-producing animals [24]. A donkey’s daily water requirement can range from 5 to 35 L [45]. However, they should generally have unlimited access to clean, fresh water [24]. Since donkeys are sensitive to water temperature, the water provided needs to be at least 15 °C [24] and never cold, because if they do not like it, they can resist thirst [24].

There is no standardized formulated feeding ration for jacks kept for reproductive purposes. Rota et al. [46] reported that donkey stallions were fed meadow hay along with commercial horse feed formulated according to NRC energy recommendation. When the jacks were kept in groups in paddocks, hay was given *ad libitum*, while when they were kept in boxes, hay and feed were provided in amounts sufficient to maintain their Body Condition Score (BCS) and weight. Although this study does not specifically address nutritional requirements, the feeding regimen was considered adequate.

The BCS is currently the primary tool used to assess feeding efficiency and to evaluate nutritional status [47]. Kovandžić et al. [48] in their welfare assessment study in dairy donkey farms reported that the animals had a BCS ranging from two to three (using 5-point scale), indicating that the donkeys were slightly thin, rather than obese. However, the optimal BCS range for dairy donkeys has not yet been defined, highlighting the need for standardization [47,49].

### 3.2. Donkey-Assisted Interventions

The relationship between humans and donkeys is long-standing, and today one of the purposes of keeping donkeys is their use in donkey-assisted interventions [50]. These interventions, including assisted activities, assisted education and assisted therapies, seek to enhance people’s quality of life [51,52]. Donkeys move slower and take more time to learn than horses, and they are also smaller with longer, softer hair. These characteristics improve tactile perception and potentially allow for more meaningful relationships. Studies have demonstrated that both donkey and horse-assisted interventions enhanced social integration and autonomy in people with intellectual disabilities, indicating that onotherapy can be an effective and valuable alternative to equine-assisted therapy. The adoption and expansion of this therapeutic approach largely depend on the policies of individual countries, which may or may not provide funding for its implementation. For instance, animal-assisted interventions are now legally recognized and controlled in Italy, where the National Health Service and private farms that provide these services occasionally work together [1]. In the UK, six Donkey-Assisted Therapy (DAT) centers are currently in operation. These facilities support a broad range of users, including at-risk youth, individuals recovering from cancer, veterans experiencing anxiety and Post-Traumatic Stress Disorder (PTSD), young people struggling with addiction, vulnerable women, and survivors of abuse and exploitation [53].

The welfare and behavioral needs of donkeys involved in donkey-assisted intervention centers are not well studied. A study conducted in the UK by Clancy et al. [53] reported that in certain donkey-assisted therapy centers, some donkeys were unwilling to participate in intervention sessions. This finding emphasizes the importance of considering individual behavior and preferences when selecting donkeys for this purpose. Even though donkeys involved in donkey-assisted interventions are not exposed to the physical stress, injury, and workload experienced by working donkeys, they must be physically and emotionally healthy to effectively participate in therapy activities [53]. A study by Panzera et al. [54] demonstrated that there were neurovegetative and ethological changes during assisted intervention sessions, emphasizing the need for physiological (e.g., heart rate variability), behavioral (e.g., ears orientation, postural changes, and behavioral complexity), and overall well-being assessment of donkeys before and during participation in such programs [55].

#### 3.2.1. Housing Systems of Donkeys Used for Donkey-Assisted Interventions

The management of donkeys involved in donkey-assisted interventions is not well documented, and there is limited information available. Donkeys are usually kept in grass-covered or sand-based paddocks, and they have a shelter for overnight access [52,56]. A recent study conducted in Italy by Sobrero et al. [52] collected information about the management of donkeys involved in animal-assisted therapy across six facilities. The donkeys lived in groups ranging from three to eight animals, in either paddocks with a small shelter (50%) or stables with a door with human-regulated access to a paddock (50%). The shelters and the stables varied significantly in structure, ranging from professionally built structures (50%) to amateur constructed solutions (50%); in all cases, the resting areas had a concrete floor covered with bedding. Sixty-seven percent of the facilities had grass-covered paddocks, while 33% were without grass. Regarding access, in 33% of cases, the paddock was available to the animals year-round, whereas in another 33%, it was regulated according to season or weather conditions. Additional shaded areas, beyond the shelter, were present in only 50% of the facilities [52].

**Figure 7:**
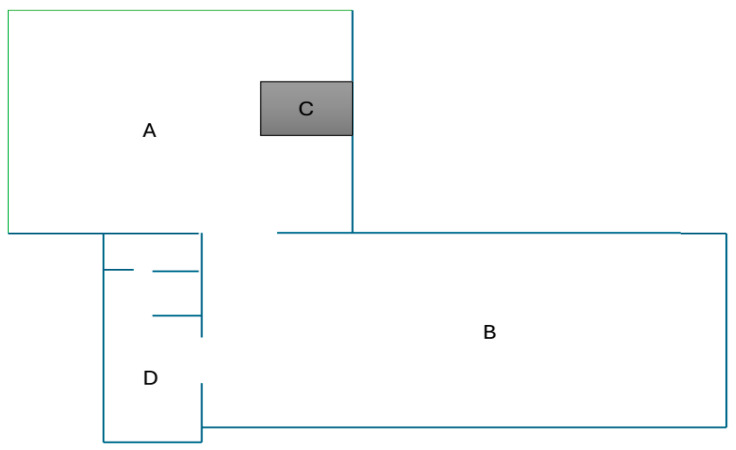
The schematic presentation of the donkey-therapy facilities found in Italy, where Lõoke et al. [56] conducted their study. Two enclosed outdoor areas linked with openings (A and B), the square food rack (C), and the stable (D). Adapted and modified from Lõoke et al. [56].

Lõoke et al. [56] described that the donkeys were kept in a group in a fenced paddock bedded with sand. The paddock included an open-sided stable with a resting area, two feeding places/mangers, an *ad libitum* water dispenser, and a salt block. The schematic presentation of the housing facilities is shown in Figure 7.

#### 3.2.2. Feeding Practices of Donkeys Used for Donkey-Assisted Interventions

The feeding practices of donkeys involved in donkey-assisted therapy do not seem different from donkeys kept for other purposes. According to Sobrero et al. [52], in most Italian facilities, donkeys were primarily fed hay. A few facilities used straw as the primary forage, with small amounts of hay provided according to the donkeys’ body weight. In addition to forages, almost all facilities provided one or more feed types. Permanent pasture was available only in a few facilities. Additionally, once a week, edible plant shrubs, fruit and vegetable servings, and cereal-based feeds (e.g., oats, barley) were provided. Fruit and vegetables were offered as a reward in most of the facilities during sessions involving visitors/users [52]. In another study, the authors reported that donkeys were fed only hay, provided in a hay rack and in several smaller piles in the paddocks [56].

Usually, the non-working donkeys that are kept in donkey-assisted facilities require feed with low energy content. This allows them to satisfy their appetite and natural foraging behavior while minimizing the risk of obesity [57,58].

### 3.3. Free-Range Donkeys

Some donkeys are living freely on the European continent, browsing and grazing [59]. Free-range (or free-roaming) donkeys usually live without human intervention and management [60]. Europe has relatively few free-roaming donkeys compared to continents such as Australia, North America, and Asia. This is largely due to agricultural policies that require donkeys to remain under some form of human management. Although a few small populations exist, they have mainly been introduced for conservation grazing purposes [60]. The selective grazing and browsing ability of donkeys is used to control and slow down the expansion of dominant grass and shrub species [59]. It has been demonstrated that this method supports insect populations and encourages the growth of endangered plant species. Donkeys are valuable in maintaining scrubby meadows and pastures that are no longer of agricultural significance [61], as demonstrated by successful programs. This method is being investigated or effectively used in a few Central European countries, including the dry meadows in the Swiss canton of Valais (Municipality of Chalais, Bavois), the former airfield in Karlsruhe (Germany), and the dry meadows in the Besançon region (France) [62].

#### 3.3.1. Housing Systems of Free-Range Donkeys

Free-range donkeys are kept on pasture without access to constructed shelters, depending on trees and other natural features of the landscape for shade and shelter [59].

#### 3.3.2. Feeding Practices of Free-Range Donkeys

Donkeys and other free-roaming equines that are employed to manage dominating grasses and shrubs typically graze all year long without the need for extra feed. According to Lamoot et al. [59], who studied the grazing behaviors of donkeys and Shetland ponies of different physiological conditions (lactating versus dry), lactating animals did not spend more time grazing than non-lactating mares in both species. However, lactating animals took more bites than dry ones, and their water requirements were met using the two pools located near the center of the reserve area [59].

### 3.4. Donkeys Used for Meat Production

Another use of donkeys that is gaining interest is meat production, with farms established in several European countries for this purpose [32,43,63]. In Italy, 5830 donkey farms are officially registered as meat farms, for a total of 21,628 animals [27].

#### 3.4.1. Housing Systems of Donkeys Used for Meat Production

**Figure 8:**
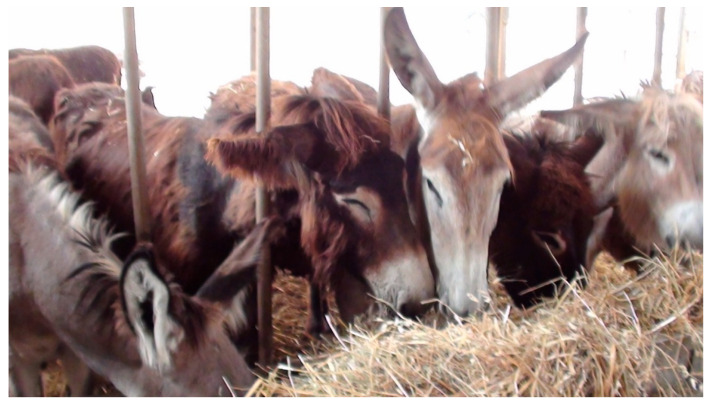
Donkeys kept in Italy in intensive systems (Padalino, picture).

In Croatia, donkeys reared for meat are kept mainly free-range and with unlimited pasture access throughout the different seasons [43]. In Italy, by contrast, some donkeys reared for meat are kept in intensive systems. They often live indoors in loose stables, where they are kept in groups in pens with limited space allowance and one common feeding area. They may have access to outdoor areas but not regularly to pasture (Figure 8) [32].

#### 3.4.2. Feeding Practices of Donkeys Used for Meat Production

Little information is available regarding feeding practices of donkeys reared for meat production [64]. Polidori et al. [64], conducted an experimental study comparing the effects of two different husbandry systems (i.e., intensive and extensive) and found that the feeding system influenced the quality of donkey meat. Animals in the intensive system group had a significantly greater final body weight than those in the extensive system group. The hot and cold carcass weight is higher in the intensive system, while protein content was greater in meat from donkeys reared extensively. Conversely, donkeys reared under the intensive system exhibited higher intramuscular fat (IMF) content. De Palo et al. [38] studied the influence of suckling techniques and slaughter age on dairy donkey meat quality and composition. They concluded that older donkeys (i.e., 18 months) who were fed artificial milk replacers had the highest IMF and protein concentrations compared to one slaughtered at 12 months who suckled naturally. In this study, donkeys were kept indoors and fed similarly after weaning. In another study, De Palo et al. [65] investigated the effect of artificial suckling on the in vivo performance and meat production traits of Martina Franca donkey foals, reporting that it has a positive impact, especially during the first six months, resulting in higher weight gain. These studies highlight the intensive nature of donkey meat production in countries like Italy, and the techniques applied to enhance meat yield [38,65]. Dai and colleagues [63] reported that donkeys for meat production in Northern Italy were kept in semi-extensive conditions; with jennies and their foals grazing freely on rotation. If needed, hay and mixed feed were provided while water was available *ad libitum.* Similarly, Polidori et al. [66] reported that foals reared for meat are kept in pasture with their dams until 6–7 months of age. After weaning, they were given polyphyte hay in the evening, while spending the rest of the day on pasture. In their study conducted in Croatia, Ivanković et al. [43] indicated that donkeys grazed on pasture and were fed hay supplemented with concentrate at 1.1% of body weight.

### 3.5. Other Donkeys (Working Donkeys, Tourism Donkeys)

The industrial revolution, agricultural mechanization and the use of vehicles for transportation of humans and various goods over the last decades have significantly reduced the use of working donkeys [1,44]. However, for example, in Greece and in parts of Spain, especially in mountainous areas and on islands, donkeys are used for tourism, forestry, and recreational needs, and occasionally for transportation of goods or people [67,68,69,70]. Additionally, in Southern and Eastern Europe, including Spain and Portugal, working equids, such as donkeys, are still used for agriculture [68,69,71].

Donkeys are also kept as pets or companion animals [72]. In the UK, people interested in adopting donkeys as companion animals can foster them through organizations, such as the Donkey Sanctuary, after completing training on donkey management. Some donkeys are also kept and managed in educational colleges, wildlife parks, and museums [72].

#### 3.5.1. Housing Systems Other Donkeys (Working Donkeys, Tourism Donkeys) 

According to Haddy et al. [69], working equids in Spain and Portugal are provided with constructed shelters in most cases (81.7%), while only 13.3% rely on natural shelter. However, the authors stated that the space provided in the shelter or stable was often insufficient. In Greece, donkeys employed in tourism are reported to lack access to shelter, water, and food during the day [68].

According to Cox et al. [72], donkeys kept as pets or companion animals in the UK are generally housed on smallholding stables, private property, or arable lands. Seasonal variations influenced the type of housing used. Most of the housing structures are open shelters providing the donkeys with free access to the outside environment. Often, there is pasture available; however, a few have only concrete flooring or a sand base floor. The shelters in most cases were bedded with straw, wood shavings, and, less commonly, rubber matting [72].

#### 3.5.2. Feeding Practices Other Donkeys (Working Donkeys, Tourism Donkeys) 

In Greece, donkeys used in tourism and for transportation of goods in remote mountainous areas were fed inadequately [67]. Haddy et al. [69] reported that pasture and hay were the main feed sources for working equids in Spain and Portugal. The study also highlighted that these animals had only limited access to water. Therefore, it is recommended that working donkeys receive supplementary feed to compensate for limited grazing time and the additional energy expenditure from work [69]. The Donkey Sanctuary recommends providing high-quality fibers, such as grass hay and alfalfa, and, if concentrate supplementation is necessary, it should be carefully regulated to avoid potential health risks [57].

Regarding donkeys kept as companion animals or pets, Cox et al. [72] reported that most of them were fed dry hay or straw, and concentrates were supplied to half of the donkeys that were included in the survey.

### 3.6. Effects of the Housing System on Welfare

The thermal neutral zone (TNZ) of an animal refers to the ambient temperature range in which core body temperature can be maintained without expending additional energy beyond that required for maintenance [73]. However, the TNZ has not been calculated for donkeys [74]. A study conducted by Proops et al. [74] found that, compared to horses, donkeys sought shelters significantly more often in cold (below 10 °C), rainy, and windy conditions. This behavior is likely related to their coat characteristics: donkeys’ coats are less protected from wet climates, as they contain less lanolin, and the hair is lighter, shorter and thinner, with minimal thickening during colder months [75]. Donkeys have been observed shivering at temperatures above the lower critical temperature suggested for horses, likely due to their adaptation to semi-arid environments. For this reason, donkeys kept in Europe are more likely to suffer from cold stress, especially if there are no shelters [74]. Shelter should always be available for donkeys when they are on pasture, and each animal should have an allocated space of at least 2 m^2^. Moreover, there must be an adequate feeding space of 90 cm length for each donkey [76]. The shelter should have a roof, at least two sides, and an opening orientated to the south in the Northern hemisphere. The back of the shelter should face north to provide protection from the prevailing cold weather [76].

Donkeys are social animals; therefore, to enhance their welfare, they should be kept in conspecific groups, with access to pasture or paddocks, allowing them to express their natural grazing and browsing behaviors [44,77]. Murray et al. [78] indicated that pair-bond and association are important in domestic donkeys. Donkeys in pairs recognize and prefer to stay close to their companion. This study suggests that keeping domestic donkeys in groups or pairs helps to avoid stress and improve their well-being and emotional state. Donkeys are rarely kept individually, except in specific cases such as housing stallions or mares close to delivery. In such cases, they may suffer from isolation stress and, therefore, should have visual and olfactory contact and interaction with other donkeys [79]. Additionally, Cox et al. [41] reported that donkeys housed individually in single boxes are at higher risk of developing impaction colic than those housed in groups. Although little is known about the optimal space allowance and group size for donkeys, the space provided and the group number should be arranged to facilitate and optimize feeding [24].

Regarding group size, wild donkeys tend to live in small groups usually no more than five individuals composed of one or more stallions, a few females, and their offspring [80,81]. In farms, the groups should not be too large, and should be kept as stable as possible; the introduction of a new female or a new male needs to be managed carefully [34]. However, there are no exact recommendations about the group size of farmed donkeys to optimize their welfare.

### 3.7. Effects of the Feeding Practices on Welfare

The African wild asses from which domestic donkeys originate were adapted to live in semi-arid climates with minimal, low-quality vegetation. As a result, donkeys have evolved to be both browsers and grazers to increase their food source [82]. When grasses and other low vegetation are scarce, they may find food in woody shrubs and trees [57,62]. Donkeys can work and survive on lower-quality feed; however, this food must not be spoiled, moldy, boiled, contaminated with soil, sludge, or wastewater [68]. In the European context, incorrect feeding is the main issue, rather than hunger and thirst [83]. Donkeys require a diet that is rich in fiber and with a low energy level. The feeding management should focus on fulfilling the natural grazing behavior of donkeys while providing the appropriate feed, preventing overweight as well as underweight [84]. Optimal milk production in donkeys can only be achieved through proper nutrition; therefore, feeding practices must be carefully managed [76]. For maintenance purposes, the daily feed intake of the donkey is 1.3–1.8% of BW in DM per day. Overfeeding causes several serious health issues, including obesity, metabolic or hormonal abnormalities, and hyperlipemia. Under any of these circumstances, an overabundance of energy stored as metabolically active adipose tissue may result in insulin resistance, enzyme dysregulation and improper fat mobilization. Overweight donkeys are more prone to develop associated diseases such as cirrhosis, laminitis, lipidosis, nephrosis, and diabetes [44]. Impaction colic is another welfare and health problem associated with an inappropriate feeding systems for donkeys. Cox et al. [41] identified paper bedding, concentrated feeding, and limited access to pasture as predisposing factors for impaction colic in donkeys [41]. The study further reported that providing extra concentrate rations increased this risk [41]. Donkeys usually do not need energy-rich cereal grains, sweet feeds, or high molasses products, and such feeding is not encouraged, as it can predispose to various health problems, including gastric ulcers, laminitis, colic, and dental disorders. If such feeds are required, starch or sugar content should not exceed 15%, with a recommended of 10% [44]. Donkeys have digestive systems adapted for continuous intake of high-fiber forage. Therefore, their feed should be offered in a distributed feeding manner throughout the day to allow sufficient time for chewing and proper digestion [82].

As suggested above, access to pasture is important. Lamoot et al. [85] investigated the grazing behavior of free-roaming donkeys and reported that during daylight hours, they spent most of their time grazing (56%). Grazing time was significantly shorter in summer (45% of the day), while the longest grazing times were achieved in spring (64%). The absence of pasture access in donkey breeding facilities affected the likelihood of having skin lesions, alopecia, low BCS, and a less positive emotional state [25].

Regarding water, Cox at al. [41] indicated that donkeys without water access are at a risk of developing impaction colic. Therefore, water should always be available, and the water bucket/troughs should be positioned at an appropriate height and location. Moreover, donkeys’ water intake should be regularly checked to keep the animals healthy and for good digestive performance [82]. Donkeys’ water requirements are similar to horses’; the quantity of water that they drink depends on the temperature, moisture content of the food, and amount of work the donkey performs [44].

### 3.8. Hoof Disorders and Care

Hoof disorders are one of the primary health issues that require regular checkups and examinations. Neglect and improper hoof management may result in lameness and chronic hoof disorders, causing pain and leading to poor quality of life [7,24,48,86]. The pain associated with hoof problems may lead to a decrease in feed intake due to the inability of the animal to move frequently to the feeding points/feed troughs [24]. The most common problems include white line abscess, seedy toe or white line disease, laminitis, fractures, keratoma, and flexural deformities [87,88].

A study conducted by Dai et al. [25] on dairy donkey farms in Italy reported that 18.7% of donkeys showed signs of hoof neglect, such as overgrowth and/or incorrect trimming. According to this study, the prevalence of hoof neglect varied by production category. Foals had the lowest risk, while jacks showed the worst hoof conditions, likely due to their limited access to pasture and the resulting lack of movement. Accordingly, signs of hoof neglect were reported in 15.16% of donkeys across 12 facilities in Italy and the UK where there was no access to pasture [6]. Cruz et al. [89] reported a high prevalence of hoof neglect (39.5%) and lameness (9.84%) in donkeys bred in Portugal, with overgrown hooves being the most common disorder. Specific hoof disorders are usually related to the type of management, environment, and work the donkey performs [87]. When donkeys are kept in wet environments, their feet absorb too much moisture and become saturated with water. This exposes the hoof to several diseases, including abscess formation, white line disease, and infections of the sole and frog (thrush) [44]. Other factors that predispose donkeys to hoof disorders are inappropriate management practices, such as feeding energy-rich diet or allowing donkeys to graze in fast-growing grass, both of which increase the risk of laminitis [88,90]. Menzies-Gow et al. [91] reported a 48.5% prevalence of laminitis among donkeys in the UK, and indicated that this condition is often recurrent, with 41.7% of animals having one or more episodes during the 3.5-year study period. Similarly, in Switzerland, laminitis and hoof abscesses were among the most frequently observed diseases by owners and veterinarians [92]. Therefore, to optimize the hoof care of non-working donkeys, a level of exercise should be mandatory.

Appropriate nutritional management is also crucial for hoof health. As reported above, feeding large amounts of energy-rich concentrate may result in laminitis [90]. Dietary change, which is associated with seasonal variations, such as when donkeys are moved from pasture back to constructed facilities, spending longer periods on hard floors, could be some of the predisposing factors [91].

Hoof care in donkeys includes daily hoof examination, and every 6–10 weeks, routine hoof trimming should be performed to avoid the development of foot problems [25]. Trimming in donkeys may be difficult due to poor handling, so it was suggested to train them to lift and hold their legs. Daily use of a hoof pick to clean the hooves facilitates careful inspection [76].

### 3.9. Dental Disorders and Care

Following hoof disorders, dental disorders are the second most prevalent health problems reported in donkeys. They represent a significant welfare concern as they are often undetected and overlooked [24,86,93,94]. The teeth should be regularly checked, and the abnormalities should be treated and corrected early before they progress to an advanced stage [86,93]. Acquired dental disorders in donkeys could be a result of an underlying developmental problem [93]. Therefore, when dealing with dental health disorders, it is essential to determine whether a developmental problem is the primary cause and address it as part of the treatment [93]. The existence of primary dental disorders can predispose donkeys to secondary acquired conditions, which complicate and exacerbate the severity of the problem [95]. The most common dental disorders in donkeys are incisors, canine teeth, and wolf teeth abnormalities (overgrowth and uneven alignment), diastemata and periodontal disease, sharp enamel points (overgrowths), displaced teeth, wear abnormalities, pulpar exposure and apical infections, cheek teeth fractures and caries, and polyodontia [94,96,97,98]. Du Toit et al. [99], performed a dental examination of 357 donkeys in the UK and reported a 73% prevalence of dental disorders, with prevalence rising to 98% in older donkeys (15–20 years) compared to only 28% in the youngest ones. The occurrence of these disorders increases with age [84,99,100], warranting prophylactic dental treatment in older donkeys (>15 years) [99]. Cruz et al. [89] reported that 62.8% of assessed donkeys require dental treatment, indicating that dental disorders may not be given the necessary attention by owners. Rodrigues et al. [101] investigated the prevalence of dental disorders in Zamorano-Leonés and Mirandês donkey breeds and reported 82.2% occurrence of dental disorders, with only 4.5% of the animals having previously received dental examination.

Dental problems are strongly linked to colic, weight loss, poor BCS, and the requirement for supplemental feeding [95]. Donkeys with dental disorders tend to have lower BCS due to the inability to masticate the available feedstuff [102]. Valle et al. [103] indicated that in lactating donkeys, animals with poor BCS have dental diseases. Cox et al. [41] in their study indicated that donkeys with dental diseases are at high risk of developing impaction colic. Similarly, Du Toit et al. [104], indicated that there is a strong association between the presence of diastemata and colic. A study, by Fernández et al. [102], explored the relationship between dental health and the management system of donkeys, and reported that inappropriate feeding practices, such as energy-rich diets in geriatric donkeys, may contribute to both obesity and dental disorders [102].

Donkeys with dental health problems often show few or no obvious symptoms, making it difficult for owners to identify them. As a result, regular periodic dental examination is important to identify dental disorders [24,93,105]. In addition to clinical examination equipment, supportive diagnostic tools such as radiography [96], computerized axial tomography [106], and non-invasive methods, like measuring faecal fibre length, can support the identification of dental disorders [107]. The recommended frequency of dental examinations for donkeys varies depending on their age group. For donkeys aged 0–5 years, dental checks should be conducted every 6 months. Between the ages of 5/15–20 years, annual examinations are generally sufficient unless abnormalities are detected. For donkeys over 15–20 years old, dental issues may be secondary to other health problems; therefore, any identified disorders should be managed continuously, and dental examinations should be performed at least every 6 months [93].

Overall, dental clinical investigation should include observation as a crucial component. Donkeys should be watched in their environment while eating their typical food [24,57,96]. For the majority of donkeys, a diet consisting of fibrous forages with restricted access to grass should be adequate and supports dental health [102,108].

## 4. Discussion

In this systematic literature review, we gathered records from major databases (Scopus and Web of Science), complemented by additional sources identified through snowballing and experts’ recommendations. Based on our database search, the number of publications addressing donkey husbandry, management, and welfare has not demonstrated a clear trend over the past 20 years as indicated in Figure 2A. Interestingly, a large proportion of these records originated from authors affiliated with institutions in Italy, the UK, Switzerland, and Portugal. This pattern may reflect the presence of strong national interests and active organizations dedicated to improving and protecting donkey welfare in these countries. Italy, for example, has emerged as a pioneer in dairy donkey production and hosts several donkey-assisted intervention centers, likely motivating Universities and research institutions to focus more on donkey related studies [1,6,21]. In the UK, the ‘Donkey Sanctuary’ one of the world’s largest non-governmental organizations dedicated to donkey welfare has significantly boosted both research and practical initiatives to improve donkey health and welfare [1,72]. The majority of the records cover general aspects of donkey biology, husbandry, and welfare. In addition, there has been a noticeable number of studies specifically focused on dairy donkeys. This reflects the growing interest in donkey milk and related products, as well as the expanding role of donkeys in therapeutic and alternative farming systems. Overall, these findings suggest that research activity on donkeys is concentrated in a few European countries with strong institutional or commercial drivers, highlighting both opportunities and gaps in the broader study of donkey welfare across Europe.

This review summarizes the main husbandry, management, and welfare issues faced by donkeys across EU MSs plus Switzerland and the UK according to their purpose and proposes recommendations to optimize their welfare. Table 2 outlines the most common production systems/husbandry types, welfare challenges, and recommendations for donkeys kept for different purposes. 

From our findings, it is apparent that welfare issues vary depending on the purpose and use for which donkeys are kept and reared [32,69,72,89]. Dairy donkeys may suffer from feeding and housing practices that are not sufficiently adapted to their specific physiological needs [6,34], while donkeys used in therapy are managed in semi-intensive systems, where frequent handling and limited enrichment raise additional concerns [56]. Working donkeys often suffer from overwork, poor nutrition, and inadequate hoof care [67,69]. Housing systems range from open shelters with outdoor access to pens with concrete or sand floors, yet few are based on evidence-based scientific recommendations. Feeding practices remain largely traditional, relying on hay and limited concentrates, often without consideration for workload or physiological state, leading to undernutrition and preventable health problems. These findings highlight the need for minimum standards for donkey care, similar to those already established for cattle, sheep, and horses. Regulations should address housing, nutrition, dental and hoof care, and species-specific needs to ensure consistent and humane management. Incorporating such standards into European frameworks would improve welfare, support sustainable donkey-based industries, and raise awareness among owners.

The recent increase in donkey populations across European countries driven by growing interest in donkey products including milk and meat, their use in cosmetics, and other purposes, such as intervention services, further underscores the necessity of improving donkey management practices. For example, the number of donkeys has slightly increased in Greece from 14,570 in 2008 to 16,443 [1]. Additionally, implementing clear regulations and standards for housing, feeding, and general welfare is essential to guarantee consistent improvements optimizing the well-being of donkeys across all production and service contexts. Donkeys were previously considered not to require welfare checkups and assessments due to their extensive management systems and relatively small population. However, this perception has shifted with the growing diversification of their use and the rise of semi-intensive and intensive management practices [83].

Housing practices for donkeys are among the areas lacking standardization and vary widely across European countries. Nonetheless, most of the donkeys kept under human care are provided with some form of shelter [48,89,92]. In Switzerland, for example, according to the assessment of Schäfer et al. [92], donkeys were typically provided run-in shelters with access to pasture; however, almost half were kept without conspecific contact. This management practice may pose a welfare concern as donkeys are social animals and need to be living in groups and forming stable social bonds [80,81]. Despite the expansion and increase in dairy donkey farms, there is still no agreement on the management and husbandry practices. Each farm generally manages and handles its donkeys according to its own experience and available resources [6,48]. Kovandžić et al. [48] assessed five different dairy donkey farms and indicated that all of them provided shelters, but the space allowance per donkey varied considerably (from 2.43 m^2^ to 16.67 m^2^), highlighting the lack of space provision standards. Some authors provided recommendations about the housing and the space allowance requirements of dairy donkeys. For example, according to Salimei and Fantuz [32] during lactation, dairy donkeys should be housed in a suitable environment, equipped with appropriate facilities. The floor should be bedded with straw and jennies with their foals should be housed together in barns or stalls. A covered feeding alley should be installed in the barn, and each jenny should have at least 4 m^2^ of indoor space for every 250 kg of weight, with an additional 3.5 m^2^ allocated for an outdoor paddock. Donkeys tend to seek shelter more than other equids, especially during low temperatures and rain. This behavior highlights that shelter and housing provisions should be mandatory for donkeys, as they require additional protection against environmental elements typical of temperate climates [74]. Other guidelines [34] developed through a collaboration between the University of Milano and the Donkey Sanctuary includes recommendations for space allowance for donkeys of different sizes [34]. The recommended shelter areas vary according to height at the withers, ranging from 5.5 to 9 m^2^ per animal for group housing and from 5.5 to 12 m^2^ for single-box housing [34]. Additionally, housing practices should consider that donkeys are social animals and should be kept in small groups rather than alone or in pairs [24]. Therefore, the space allowance and social grouping of donkeys requires further study to better address their species-specific needs.

The feeding and nutritional requirements of donkeys are another key area lacking detailed studies and standards [24]. The feeding of donkeys kept for various purposes is generally not based on scientifically formulated diets. Instead, it is mostly guided by traditional practices within the donkey-rearing community, relying primarily on forage such as hay with the addition of concentrate supplements. Most of the donkey population in European countries, such as the UK, suffers from being overweight and obese, especially those kept as pets or companion animals [58,90]. Obesity predisposes donkeys to different health problems such as hyperlipemia, osteoarthritis, and laminitis [58,90]. In Switzerland, Schäfer et al. [92] reported that one–third of the assessed donkeys were suffering from being overweight, a condition related to their husbandry, management, and age. On the other hand, lactating donkeys are often reported to be thinner, with lower BCS and body weight [48], which may predispose them to conditions such as hyperlipemia [42]. Poor body condition caused by an unbalanced diet or inadequate feeding may lead to intestinal diseases and negatively impact donkey welfare, including reduced milk production and overall productivity [83]. It is, therefore, important to emphasize that the feeding and nutritional requirements of donkeys used for different purposes should be studied in detail to prevent the occurrence of obesity, unnecessary weight loss, and disease conditions, ultimately improving their welfare [24,58].

Dental health and hoof problems are among the major welfare issues reported in donkeys found in the investigated countries [25,86,89,91,92,93]. Dental problems could often be associated with colic, weight loss, and poor BCS, which consequently increases the need for supplemental feeding [95]. Appropriate feeding management [102,108], regular dental examination, and timely treatment of abnormalities are crucial to improve the welfare of donkeys and should, therefore, be mandatory [86,93]. Neglect and improper hoof management can lead to lameness and chronic hoof conditions, significantly compromising donkey welfare by causing pain [6]. Inappropriate feeding, lack of exercise, wet environments, obesity or being overweight, hard floors, and digestive problems are some of the factors predisposing donkeys to hoof disorders [48,58,87]. Regular hoof examination, treatment and trimming of overgrown hooves is important to prevent these issues [25]. The authors believe that hoof problems and dental disorders represent the primary health issues that negatively affect donkey welfare. Without proper attention and management, these issues can cause significant pain, discomfort, and secondary health complications, ultimately diminishing the overall well-being of donkeys.

Additionally, assessing donkeys’ well-being through standardized protocols is essential to further improve welfare and enhance their overall quality of life. Acknowledging the welfare challenges faced by donkeys, the Animal Welfare Indicators (AWIN) project, developed a protocol for assessing the welfare of donkeys on farms [49]. The protocol was developed considering the four welfare principles [109]. This protocol has been successfully applied in assessing the welfare of donkeys under different farming and management conditions [48,89]. However, larger studies assessing more farms using standardized welfare protocols are needed to classify the farms and quantify objectively the level of welfare of donkeys kept for milk and other purposes in EU MSs and the UK.

When interpreting the results, it is important to take into account the limitations of this review. Firstly, the literature search was limited to the Scopus and Web of Science databases which may have excluded relevant studies available in other sources. Furthermore, the number of possibly pertinent documents may have been further decreased by applying specified filters, such as restricting the language to English, concentrating on specific scientific areas, specific countries of the corresponding authors, and including only specific document types. Finally, even though the authors carefully selected the keywords for the search, it is possible that some relevant details were inadvertently missed, leading to the exclusion of potentially relevant research. Other limitations include the omission of vaccination, and deworming practices, which are topics that need to be considered in other studies, as not addressed in this review. Despite these limitations, we have made a comprehensive effort to identify and discuss the common management, husbandry, and welfare issues affecting donkeys. Based on our literature review, donkeys are managed in a variety of ways, with housing and shelter types varying depending on their intended use. However, the grouping and housing arrangements commonly used are often not supported by scientific evidence and may fail to meet the behavioral and natural needs of donkeys. Additionally, feeding practices in the donkey-rearing community are largely traditional and often lack a scientific basis. Furthermore, consistent access to pasture is often lacking. This management approach may lead to overweight donkeys and related health problems, and in some cases, may fail to meet their physiological and production requirements. Therefore, both housing and feeding practices represent key areas where further research is needed to support improvements in donkey management, husbandry, and overall welfare. Additionally, dental and hoof disorders remain among the most common health concerns for donkeys. These issues are frequently associated with inadequate management, aging, and limited opportunities for natural behaviors such as grazing, browsing, and regular movement. Ensuring regular dental and hoof care is essential to prevent these conditions and to enhance the health, welfare, and overall quality of life of donkeys.

## 5. Conclusions

Our review provides important information regarding the existing management and husbandry practices of donkeys across the EU MSs, Switzerland, and the UK. Additionally, it has identified key gaps that need to be addressed through targeted studies, highlighting the areas lacking sufficient research in donkey production, management, and welfare. Donkeys are managed differently based on their use and intended purpose. They are more likely than other equids to seek shelter, especially in cold or rainy conditions, highlighting the importance of appropriate housing and suitable group sizes given their social nature. Providing adequate space and properly sized groups is also essential to facilitate feeding and prevent overcrowding. In the countries investigated, most donkeys suffer from being overweight, although there are some reports of thinner lactating donkeys exhibiting lower body weight. These findings highlight that, in many cases, donkey feeding practices are not guided by scientific principles. Therefore, housing and feeding management of donkeys should be studied in detail to ensure that nutrition is tailored to their intended use and purpose. Other major issues affecting donkey welfare include dental and hoof disorders, which are often linked to poor management. Regular dental and hoof examinations are essential to detect abnormalities early and provide timely treatment before these problems worsen and negatively impact the donkeys’ well-being.

## Figures and Tables

**Table 1:** Exclusion and inclusion criteria applied to screened records.

Inclusions	Exclusions
- Studies focusing on the management and husbandry of donkeys in EU MSs, Switzerland, and the UK	- Studies not conducted in EU MSs, Switzerland, and the UK
- Studies on the welfare assessment of donkeys in EU MSs, Switzerland, and the UK	- Studies exclusively dealing with donkey milk quality and composition
- Studies on the dental disorders/care and their welfare consequences in donkeys in EU MSs, Switzerland, and the UK	- Studies exclusively dealing with donkey milk technology, such as the preservation of donkey milk or cheese formation from donkey milk
- Studies on hoof disorders/care and their welfare consequences in donkeys in EU MSs, Switzerland, and the UK	- Studies exclusively dealing with the nutritional and health values of donkeys’ milk
- Studies on welfare issues and concerns in the donkey industry	- Studies on specific feed type nutritional composition and its impact on meat or milk production and quality nutrition
	- Studies on the donkey meat quality
	- Studies on different species, such as horses, mules

EU MSs: European Union member states; UK: United Kingdom.

**Table 2:** Summary of the most common production system/husbandry, welfare challenges, and recommendations for donkeys kept for different purposes.

Purpose of Donkey Keeping	Main Type of Husbandry (Production Systems)	Welfare Challenges	Recommendations
Dairy donkey	– Donkeys are managed under extensive, semiextensive, and semiintensive husbandry systems [2,20,21,23,30,31] – They are kept on pasture with a constructed shelter [21]	– Lack of a separate shelter for overnight access [21] – Limited understanding of the nutritional requirements of dairy donkeys at different stages of production, such as pregnancy and lactation [24] – Lack of specific formulated ratios for lactating and nonlactating jennies [23]	– Detailed studies are needed to understand the nutritional requirements of a dairy donkey and their intake – Provision of access to appropriate daytime and overnight shelters – Provision of pasture access to practice natural behavior such as grazing and browsing
Donkey-assisted interventions	– Donkeys are kept on grass or sandbased paddocks, and they have a shelter for overnight access [52,56]	– Experience lack of interest in participating in intervention sessions [53] – Lack of access to pasture [56]	– Donkeys should be emotionally healthy to effectively participate in intervention and therapy activities [53] – Individual behavior and preferences should be taken into consideration when selecting donkeys
Free-range donkeys	– Donkeys live on pasture without human intervention and management [60] – Donkeys live without constructed shelters, and they depend on trees and other natural features of the landscape for shade and shelter [59]	– Exposed to extreme weather conditions [59]	– Donkeys should be provided with natural or constructed shelter, especially during extreme weather
Donkeys used for meat production	– Donkeys are kept mainly with unlimited pasture access throughout the different seasons [43] – In countries such as Italy, by contrast, donkeys are kept in intensive systems, where they live in groups [32]	– Lack of appropriate shelter [43] – No regular pasture access in the intensive production system [32]	– Donkeys in the extensive system, should be provided with the appropriate shelter – Detailed studies on the nutritional requirements and feeding practices of donkeys intended for meat production are needed
Working and tourism donkeys	– In Spain and Portugal donkeys are provided with constructed shelters in most cases, while a small proportion relied on natural shelter [69] – In Greece, they are kept open without any kind of shelter [68]	– Insufficient space provided in the shelter or stable [68,69] – Lack of shelter [69] – Lack of water and food during the daytime [69] – Inadequate feeding [67]	– Provision of appropriate housing – Professional guidance on the nutritional needs of working donkeys [67]
Companion donkeys	– Donkeys are kept in various housing types depending on the season [72] – Mostly kept in open shelters with free outdoor access, usually with pasture, though some have only concrete or sand flooring [72]	– Lack of pasture access [72] – Absence of conspecific groups	– Donkeys should be kept in conspecific groups – Provision of pasture

## Data Availability

The data presented in this study can be requested to the corresponding author.

## Supplementary Materials

The following supporting information can be downloaded at: https://www.mdpi.com/article/10.3390/ani15192768/s1, Table S1: The different keywords and final search string used in Scopus and Web of Science databases to extract the relevant documents. Table S2: The different filters used in Scopus and Web of Science databases to extract the relevant documents.
