# Supplementary material for: Systematic Literature Review on Donkeys (Equus asinus): Husbandry and Welfare in Europe
Source: Animals (Basel). 2025 Sep 23;15(19):2768. doi: 10.3390/ani15192768 (PMC12523398; doi:10.3390/ani15192768)
Supplement: Supplementary file 1 [file animals-15-02768-s001.zip › animals-3844761-supplementary.pdf]

Supplementary Tables

Supplementary Table S1. The different keywords and final search string used in Scopus and Web of Science databases to extract the relevant documents.

Database	Keywords combinations used for the search
**Scopus**	were (donkey* OR "*Equus asinus*") AND TITLE-ABS-KEY (welfare OR husbandry OR (hous* W/4 (system* OR condition*)) OR housing OR (feed* W/4 (system* OR practice* OR management)) OR (nutrition W/4 (system* OR practice* OR management)) OR (management W/4 (system* OR practice*)) OR milk OR meat OR stabl* OR shelter* OR shade OR barn OR stall* OR hoof OR hooves OR trimming OR farriery OR dental OR teeth OR oral)
**Web of Science**	TS=(donkey* OR "*Equus asinus*") AND TS=(welfare OR husbandry OR (hous* NEAR/4 (system* OR condition*)) OR housing OR (feed* NEAR/4 (system* OR practice* OR management)) OR (nutrition NEAR/4 (system* OR practice* OR management)) OR (management NEAR/4 (system* OR practice*)) OR milk OR meat OR stabl* OR shelter* OR shade OR barn OR stall* OR hoof OR hooves OR trimming OR farriery OR dental OR teeth OR oral).

Supplementary Table S2. The different filters used in Scopus and Web of Science databases to extract the relevant documents

Database	Filter criteria			
	**Years**	**Language**	**Research areas**	**Country of Affiliation**
**Scopus**	2005-2025	English	Veterinary, Agricultural and Biological Sciences	(LIMIT-TO (AFFILCOUNTRY , "Italy") OR , "United Kingdom") OR, "Spain") OR, "France") OR, "Portugal") OR, "Belgium"), "Netherlands") OR"Germany") OR, "Greece") OR, "Poland") OR, "Cyprus") OR, "Sweden") OR, "Romania") OR, "Ireland") OR, "Austria") OR Norway") OR Switzerland") OR , "Denmark") OR , "Croatia") OR , "Czech Republic") OR , "Bulgaria") OR Hungary") OR , "Finland") OR , "Slovenia") OR , "Slovakia"))
**Web of Science**	2005-2025	English	Veterinary Sciences; Agriculture	ITALY or ENGLAND or SPAIN or FRANCE or SCOTLAND or GERMANY or PORTUGAL or BELGIUM or ROMANIA or NETHERLANDS or GREECE or IRELAND or DENMARK or POLAND or SWEDEN or CYPRUS or SWITZERLAND or CZECH REPUBLIC or CROATIA or AUSTRIA or NORWAY or SLOVENIA or HUNGARY or WALES or BULGARIA or FINLAND or LUXEMBOURG or NORTH IRELAND
